# Cloning, Characterization, and Functional Investigation of *VaHAESA* from *Vitis amurensis* Inoculated with *Plasmopara viticola*

**DOI:** 10.3390/ijms19041204

**Published:** 2018-04-16

**Authors:** Shaoli Liu, Chi Zhang, Nan Chao, Jiang Lu, Yali Zhang

**Affiliations:** 1 The Viticulture and Enology Program, College of Food Science and Nutritional Engineering, China Agricultural University, Beijing 100083, China; shaolil@126.com (S.L.); cauzhangchi@163.com(C.Z.); 2 Center for Plant Biology, TSinghua University, Beijing 100084, China; chaonan1989@126.com; 3 Center for Viticulture and Enology, School of Agriculture and Biology, Shanghai Jiao Tong University, Shanghai 200024, China; jiang.lu@sjtu.edu.cn; 4 Guangxi Crop Genetic Improvement and Biotechnology Laboratory, Guangxi Academy of Agricultural Sciences, Nanning 530007, China

**Keywords:** downy mildew, grapevine, PRRs, PTI, *VaHAESA*

## Abstract

Plant pattern recognition receptors (PRRs) are essential for immune responses and establishing symbiosis. Plants detect invaders via the recognition of pathogen-associated molecular patterns (PAMPs) by PRRs. This phenomenon is termed PAMP-triggered immunity (PTI). We investigated disease resistance in *Vitis amurensis* to identify PRRs that are important for resistance against downy mildew, analyzed the PRRs that were upregulated by incompatible *Plasmopara viticola* infection, and cloned the full-length cDNA of the *VaHAESA* gene. We then analyzed the structure, subcellular localization, and relative disease resistance of *VaHAESA*. *VaHAESA* and PRR-receptor-like kinase 5 (RLK5) are highly similar, belonging to the leucine-rich repeat (LRR)-RLK family and localizing to the plasma membrane. The expression of PRR genes changed after the inoculation of *V. amurensis* with compatible and incompatible *P. viticola*; during early disease development, transiently transformed *V. vinifera* plants expressing *VaHAESA* were more resistant to pathogens than those transformed with the empty vector and untransformed controls, potentially due to increased H_2_O_2_, NO, and callose levels in the transformants. Furthermore, transgenic *Arabidopsis thaliana* showed upregulated expression of genes related to the PTI pathway and improved disease resistance. These results show that *VaHAESA* is a positive regulator of resistance against downy mildew in grapevines.

## 1. Introduction

Downy mildew is caused by the oomycete *Plasmopara viticola* and is one of the major diseases affecting grapevines worldwide. However, grapevines possess natural resistance against *P. viticola* as a result of disease resistance synergy. These resistance mechanisms involve physiological, ecological, and morphological changes in the plant [1,2]. An important mode of active defense in plant immunity is the detection of pathogen-associated molecular patterns (PAMPs) by pattern recognition receptors (PRRs) [3], otherwise known as PAMP-triggered immunity (PTI) [4,5]. PTI comprises a wide array of responses, including the rapid generation of reactive oxygen species (ROS), deposition of callose, activation of mitogen-activated protein kinases (MAPKs), and expression of immune-related genes [3,5]. Consequently, PTI plays a major role in preventing the pathogenic invasion of plants.

Plant PRRs are either receptor-like kinases (RLKs) or receptor-like proteins (RFLPs), taking the form of single-pass transmembrane proteins with extracellular domains. While RLKs have an intracellular kinase domain, RFLPs lack this cytosolic signaling domain [6]. PRRs exhibit both high sensitivity to and specialization for plant pathogens, where a certain PRR can recognize PAMPs at nanomolar quantities [4], and conserved functional domains of PAMPs are often recognized by PRRs. For example, FLS2 is one of the best-studied plant PRRs in *Arabidopsis* and recognizes bacterial flagellin via perception of the conserved 22-aminoacid epitope flg22 [7]. However, while FLS2 is highly conserved in plant species, the FLS2 homolog of tomato (*LeFLS2*) recognizes the flg15 polypeptide of *Escherichia coli* but does not recognize *Pseudomonas syringae* flg22 [8]. It has been hypothesized that the ligand-induced endocytosis and degradation of FLS2 may regulate receptor signaling [9]. In addition, the elongation factor Tu (EF-Tu) receptor (EFR) is a PRR in *Arabidopsis* that recognizes EF-Tu from bacteria [10]; however, this kind of PRR exists only in cruciferous species [11]. At present, the complete genome sequences of plants that contain homologous EFR genes are not well characterized [3].

Although the PAMPs of pathogenic microorganisms have been extensively studied, few of these studies have focused on the corresponding PRRs in plants. Furthermore, there are relatively few reports regarding the PRRs of grapevines. With advances in whole-genome sequencing, research focusing on characterization of resistance genes in grapevines is increasing, and recently published transient expression assays have been widely used for the characterization of newly discovered genes, including their functions and metabolic pathways [12,13,14]. Transcriptomic and proteomic analyses of grapevines infected with *P. viticola* are likely to result in the discovery of novel genes involved in pathways related to resistance against downy mildew, in addition to helping to elucidate the molecular mechanisms involved in the resistance response.

The present study describes the novel *V. amurensis* PRR gene *VaHAESA*. This gene was identified by analyzing the transcriptome of the *V. amurensis* cultivar “Shuanghong” while infected with either the compatible *P. viticola* strain “ZJ-1-1” or the incompatible *P. viticola* strain *“*JL-7-2” [13]. Here, we demonstrate that *VaHAESA* belongs to the LRR-RLK (leucine-rich repeat receptor-like protein kinase) family of proteins. Transient expression studies indicate that *VaHAESA* can trigger a series of PTI responses, including the accumulation of H_2_O_2_ and NO as well as the deposition of callose. This research provides a better understanding of the characteristics and function of a novel PRR gene in grape.

## 2. Results

### 2.1. PRR Expression in Vitis amurensis ‘Shuanghong’ Infected with Incompatible and Compatible Strains of Plasmopara viticola

**Figure 1:**
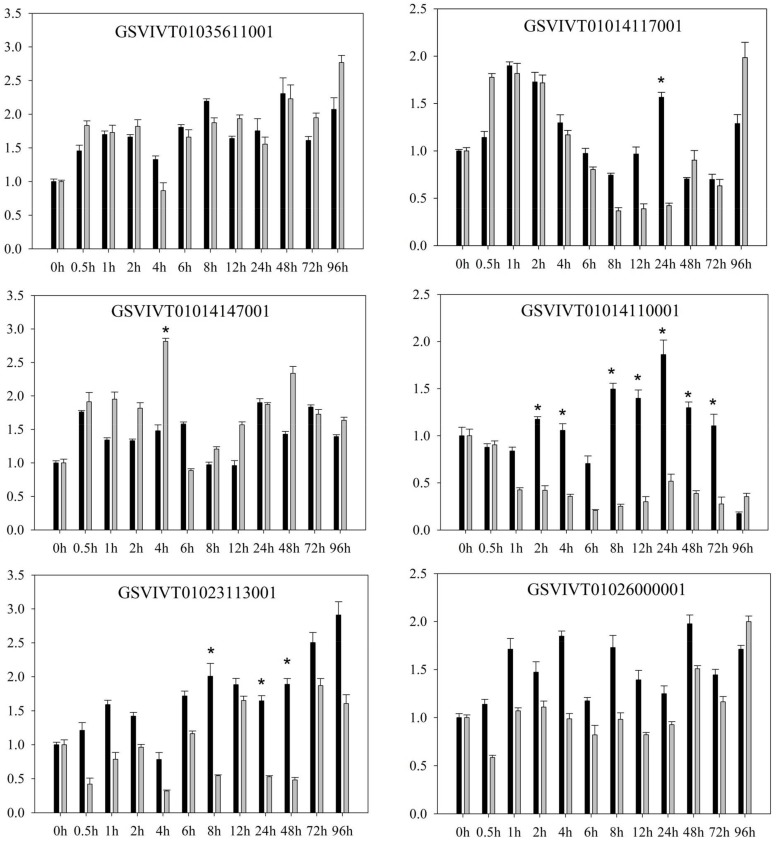
Relative expression of the pattern recognition receptors (PRR) genes of *Vitis amurensis* “Shuanghong” after inoculation with *Plasmopara viticola* “ZJ-1-1” (black bars) and *P. viticola* “JL-7-2” (gray bars). The values on the vertical axes indicate the fold-changes in gene expression normalized to the expression level of Vitis elongation factor 1-α (EF1-α), SAND, and ubiquitin-conjugating enzyme (UBQ). The x-axes represent the time since inoculation. The error bars represent the standard deviation calculated from three replicates. * indicates significant differences (*p* < 0.05) as determined with Student’s *t*-test. Significant differences were identified by comparing the two gene expression levels at each time point.

**Figure 2:**
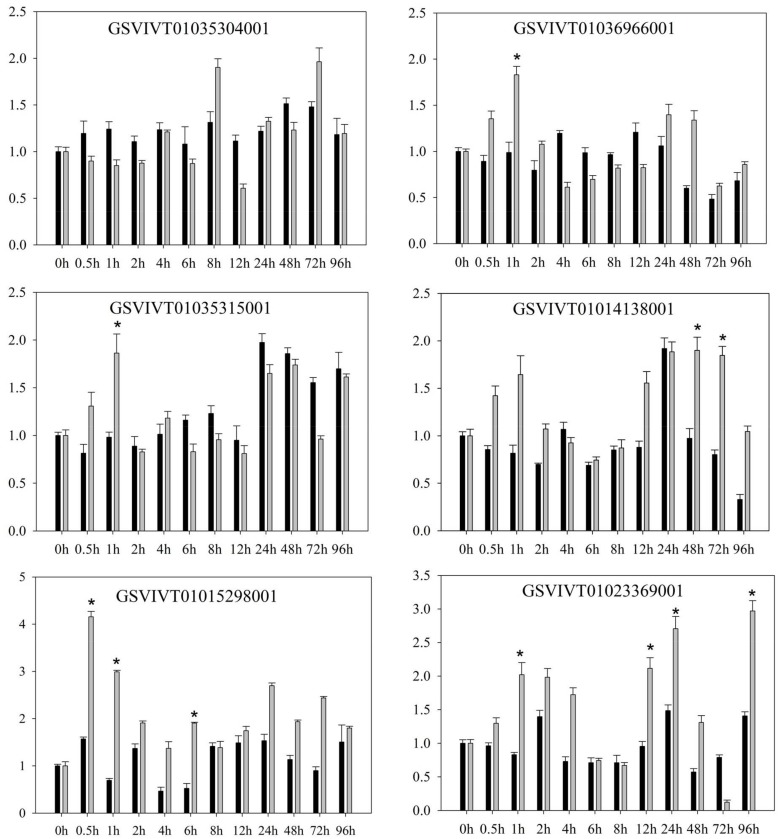
Relative expression of the PRR genes of *Vitis amurensis* ‘Shuanghong’ after inoculation with *Plasmopara viticola* “ZJ-1-1” (black bars) and *P. viticola* “JL-7-2” (gray bars). Values on the vertical axes indicate the fold-change in gene expression, normalized to the expression level of Vitis EF1-α, SAND, and UBQ. The *x*-axes represent the time since inoculation. Error bars represent the standard deviation calculated from three replicates. * indicates significant differences (*p* < 0.05) as determined with Student’s *t*-test. Significant differences were identified by comparing the two gene expression levels at each time point.

Quantitative RT-PCR revealed several differences in the expression patterns of PRR genes in *V. amurensis* “Shuanghong” (Figure 1 and Figure 2) after inoculation with compatible (“ZJ-1-1”) and incompatible *P. viticola* (“JL-7-2”) strains. After inoculation with either compatible *P. viticola* “ZJ-1-1” or incompatible *P. viticola* “JL-7-2”, clear and consistent trends were observed in the expression (both up- and downregulation) of some genes. These affected genes included GSVIVT01035611001, GSVIVT01014117001, and GSVIVT01014147001. For example, the expression of GSVIVT01014147001 increased 2-fold within 0.5 h after inoculation with the *P. viticola* strains “ZJ-1-1” and “JL-7-2”. In contrast, some PRRs were upregulated after inoculation with *P. viticola* “ZJ-1-1” but downregulated after inoculation with incompatible *P. viticola* “JL-7-2” (i.e., GSVIVT01035304001, GSVIVT01026000001, GSVIVT01014110001, and GSVIVT01023113001).

However, among the remaining genes, we observed downregulation after inoculation with “ZJ-1-1” and upregulation after inoculation with ‘JL-7-2’ (i.e., GSVIVT01036966001, GSVIVT01035315001, GSVIVT01014138001, GSVIVT01015298001, and GSVIVT01023369001). Within 0.5 h of inoculation with JL-7-2, the expression of all PRR genes that we evaluated was initially induced and then decreased. GSVIVT01015298001 was upregulated 4-fold, while GSVIVT01036966001, GSVIVT01035315001, GSVIVT01014138001, and GSVIVT01023369001 were upregulated 2- to 3-fold. In addition, the expression levels of these five genes indicated inhibition within the first 12 h of “ZJ-1-1” inoculation. According to these results, we hypothesize that GSVIVT01036966001, GSVIVT01035315001, GSVIVT01014138001, GSVIVT01023369001, and, most notably, GSVIVT01015298001 play an active role in pathogen resistance during the early phase of resistance against *P. viticola* infection. Next, based on the results of expression analyses, we chose the PRRs with the potential to confer stronger resistance against downy mildew in ‘huanghong’ (i.e., upregulated genes showing significant differences after inoculation with incompatible *P. viticola* and downregulated genes showing significant differences after inoculation with the compatible strain). Thus, we chose GSVIVT01015298001 for further functional verification of *P. viticola* resistance in *V. amurensis* “Shuanghong”, which was expressed at the earliest post-inoculation time point (0.5 h) and exhibited maximum upregulation.

### 2.2. Characterization and Phylogenetic Analysis of VaHAESA

**Figure 3:**
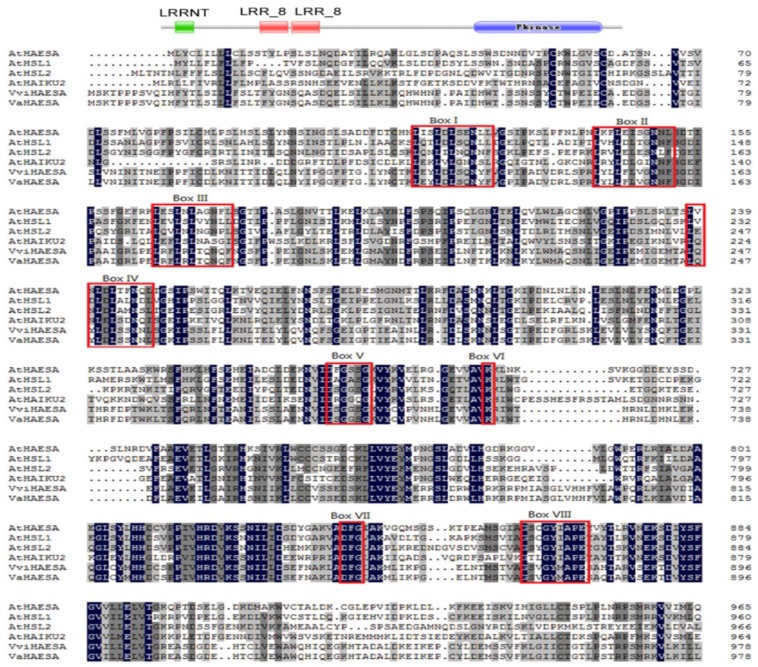
Alignment of the putative *Vitis amurensis* HAESA (*VaHAESA*) protein sequence with other HAESA and HAESA-like proteins. Box I–IV: leucine-rich repeat (LRR) core LXXLXLXXNXL motifs; Box V–VII: motifs involved in the kinase catalytic core.

We cloned *VaHAESA* from the “Shuanghong” subspecies of grape based on the aforementioned transcriptome data. The *VaHAESA* gene is 3066 bp long and encodes 1021 amino acids. Further characterization of the *VaHAESA* protein sequence using the Pfam and SUPERFAMILY 2 databases confirmed that *VaHAESA* belongs to the LRR-RLK protein family, due to the presence of a leucine-rich repeat N-terminal domain (LRRNT), two LRR_8 domains, and a protein kinase C-terminal domain. These findings are consistent with a sequence alignment and phylogenetic analysis using putative *VaHAESA* and RLK homologs from a variety of plant species (Figure 3). We detected several LRR core motifs (Figure 3, Box I–IV); these LRR repeats constitute a novel class of α/β folds. The LRR core (LXXLXLXXNXL) motifs, which form an α/β sheet, are thought to form an exposed face involved in protein-protein interactions [15]. In addition, we detected the key motifs constituting the catalytic core of the kinase. These motifs include (i) GXGXXG (Figure 3, box V), which is thought to be an integral part of many nucleotide-binding proteins; (ii) a highly conserved Lys amino acid (Figure 3, box VI); and (iii) a DFG(Asp-Phe-Gly) motif (Figure 3, box VII). Finally, the conserved motif G(T/S)PXYXAPE (Figure3, box VIII) is characteristic of serine/threonine kinases [15].

**Figure 4:**
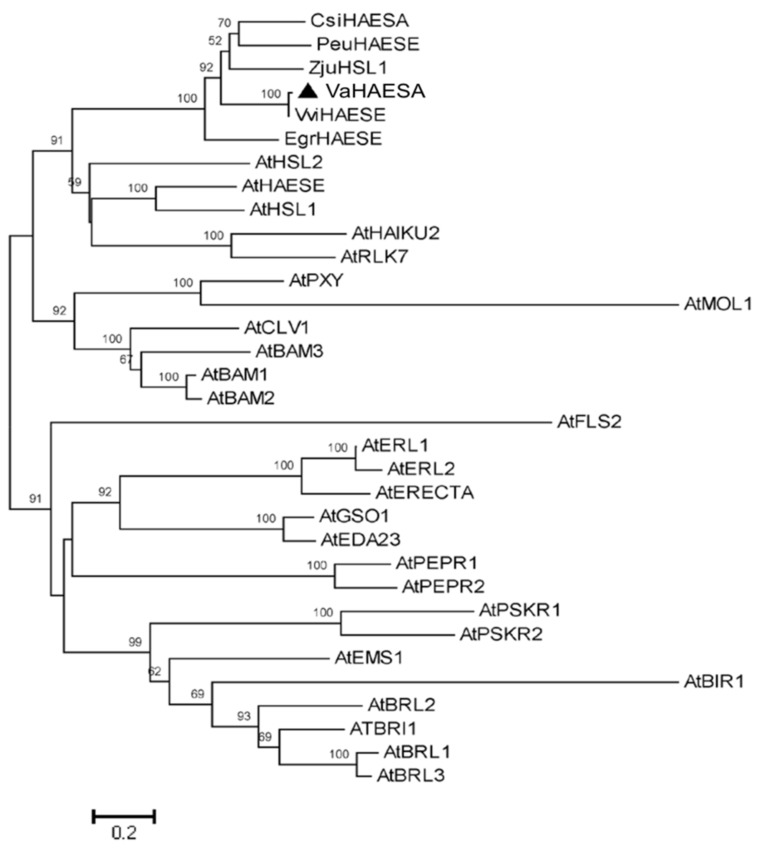
Phylogenetic tree of LRR-RLKs. *Vitis amurensis* HAESA (*VaHAESA*) is indicated by the filled triangle.

**Figure 5:**
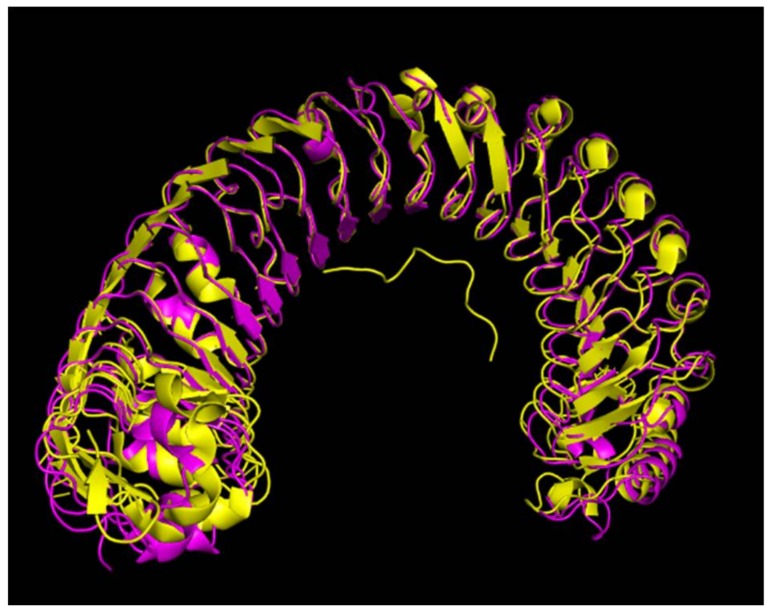
Alignment of the 3-D structures of *VaHAESA* and *Arabidopsis thaliana* HAESA *(AtHAESA)* (5IXQ).

A phylogenetic analysis revealed that the LRR-RLK that we identified in the present study (*VaHAESA*) clustered with HAESA and HAESA homologs from *Arabidopsis* and five other species (Figure 4). These results further confirmed that this gene is likely to be the *HAESA* gene of *V. amurensis* ‘Shuanghong’. HAESA, which has also been called RLK5, is a critical component required for floral organ abscission, belonging to LRR XI based on the classification of LRR-RLKs [16]. A homology matrix for these sequences is provided as supplemental material. In addition, we also established a model of *VaHAESA* based on the reported crystal structure for HAESA from *Arabidopsis* (5IXQ) (Figure 5). The crystallization data for PDB 5 IXQ are shown in yellow and correspond to the crystal structure of the *Arabidopsis* receptor kinase HAESA LRR ectodomain. The crystal structure of *VaHAESA* is shown in magenta and was obtained by Swiss-model homology. The comparison of the alignment results shows that the overall structures are very similar. The details are shown in Figure 5.

### 2.3. Subcellular Location of VaHAESA in Nicotiana benthamiana

**Figure 6:**
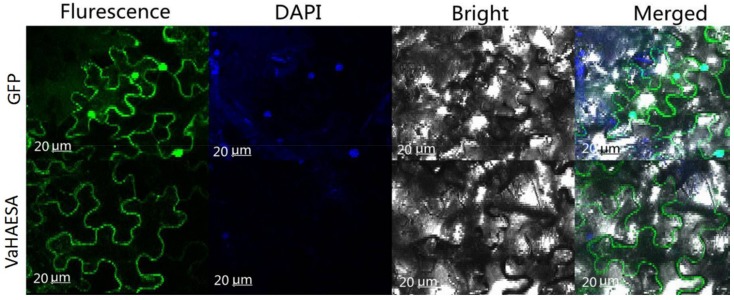
Subcellular localization of the *GFP*-*VaHAESA* protein in transiently transformed *Nicotiana benthamiana*.

Based on the differences in expression observed for the PRRs of *V. amurensis*, we selected *VaHAESA* for further investigation. To study the distribution and cellular localization of *VaHAESA* in the mesophyll cells of *N. benthamiana*, we designed green fluorescent protein-tagged constructs of *VaHAESA* downstream of the signal peptide cleavage site. The fusion constructs were expressed transiently in *N. benthamiana* using Agrobacteria infiltration. The results showed that the *VaHAESA* protein was only observed in the cytoplasm (Figure 6).

### 2.4. Expression of VaHAESA Promoted Resistance against Plasmopara viticola in Grapevine

**Figure 7:**
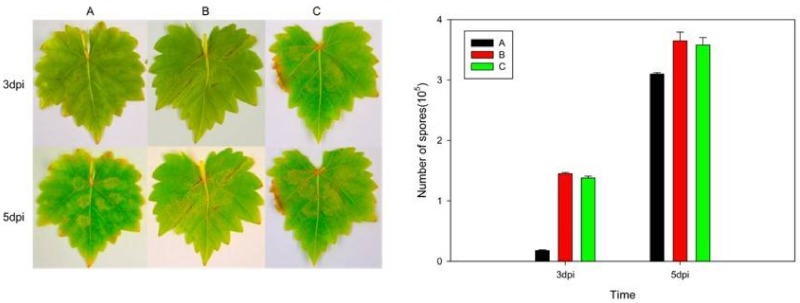
Phenotypes of leaves from *Vitis vinifera* after infection with *Plasmopara viticola*. (**A**) *V. vinifera* expressing *VaHAESA*; (**B**) *V. vinifera* transformed with the empty vector (pBI121); (**C**) untransformed *V. vinifera* “Thompson Seedless”. The representative images were taken three and five days post-infection (dpi) with *Plasmopara viticola*.

The transient expression analysis showed that 3 d after inoculation, grape leaves transiently transformed with the *VaHAESA* construct exhibited smaller infected areas than those transformed with the empty vector and untransformed *V. vinifera* “Thompson Seedless” (Figure 7). The number of spores on the *VaHAESA*-expressing plants was 0.18 × 10^5^, whereas those on the empty vector-transformed plants and untransformed controls were 1.45 × 10^5^ and 1.38 × 10^5^, respectively. These results show that disease resistance was improved accordingly in the transient grape leaves. However, five days after inoculation, the differences in the infected areas and spore concentrations on the leaves from all groups decreased (Figure 7).

**Figure 8:**
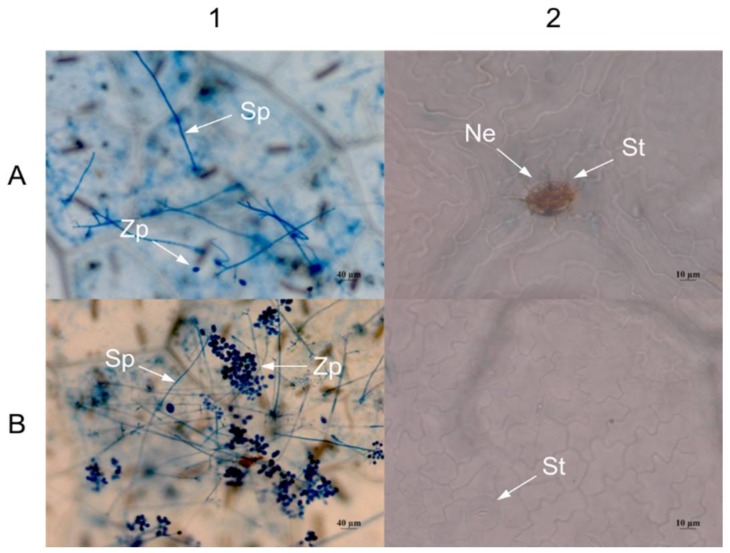
Microscopy examination of *Vitis vinifera* after infection with *Plasmopara viticola*. (**A**) *V. vinifera* expressing *VaHAESA*; (**B**) *V. vinifera* transformed with the empty vector (pBI121). Representative images were taken 3 days after inoculation with *Plasmopara viticola*. Sp: Sporophore; Zp: Zoospore; St: Stomata; Ne: Necrosis.

Three days after inoculation, the density of sporophores and spores in grape leaves transiently transformed with *VaHAESA* was lower than that in leaves transformed with the empty vector (Figure 8A1,B1). In addition, we observed necrosis in the guard cells of grape leaves transiently transformed with the *VaHAESA* gene (Figure 8A2), whereas the leaves transformed with the empty vector did not present similar necrosis (Figure 8B2).

### 2.5. Measurements of H_2_O_2_, NO, and Callose in Vitis vinifera Transiently Expressing VaHAESA

**Figure 9:**
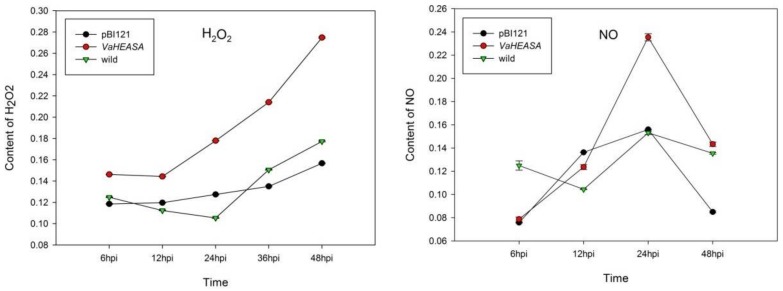
Analysis of H_2_O_2_ and NO levels in *Vitis vinifera* after inoculation with *Plasmopara viticola*.

We observed that 24 h after infection in *V. vinifera*, the H_2_O_2_ and NO contents of leaves transformed with *VaHAESA* were higher than those of leaves transformed with the empty vector or the untransformed control (Figure 9).

**Figure 10:**
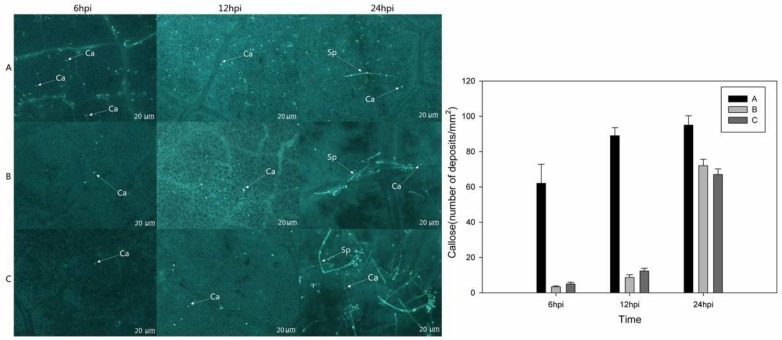
Microscopic assessment and statistics of callose deposition in the leaves of *Vitis vinifera* after inoculation with *Plasmopara viticola*. (**A**) *V. vinifera* expressing *VaHAESA*; (**B**) *V. vinifera* transformed with the empty vector (pBI121); (**C**) untransformed *V. vinifera* ‘Thompson Seedless’. Representative images were taken 6, 12, 24, and 48 h post-infection (hpi). Ca: Callose, Sp: sporophore.

Microscopic observations indicated that after inoculation with *P. viticola*, callose formation at 6 hpi and 12 hpi was much greater in the *VaHAESA*-expressing leaves than in leaves transformed with empty vector and untransformed *V. vinifera* (Figure 10). In addition, we observed few sporophores on leaves transformed with *VaHAESA* at 24 hpi, while a mass of sporophores was observed on the leaves transformed with the empty vector and wild Thompson Seedless (untransformed control).

### 2.6. Identification and Analysis of Disease Resistance in Transgenic Arabidopsis thaliana

**Figure 11:**
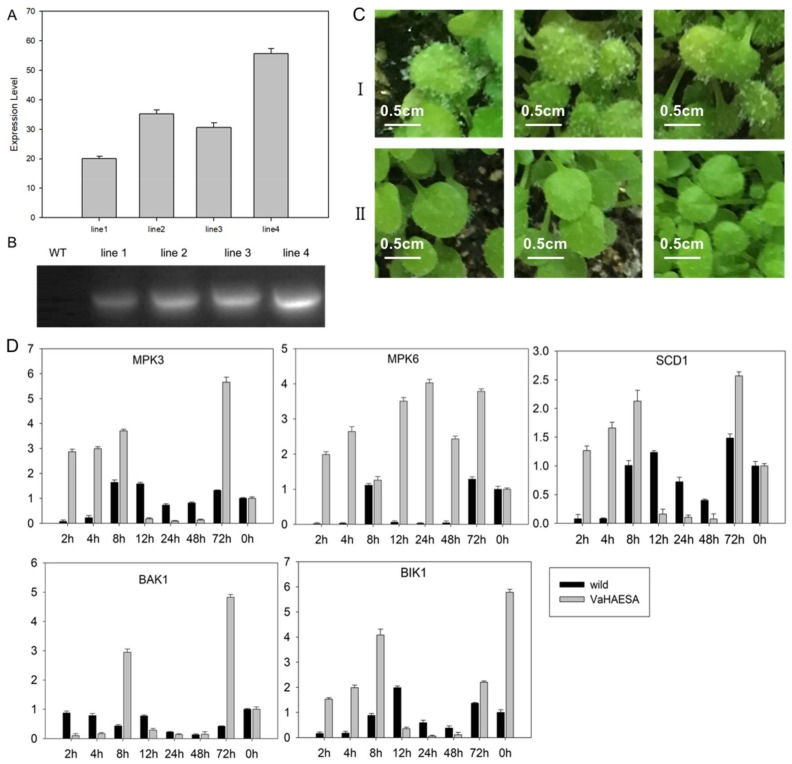
Identification of disease resistance in transgenic *Arabidopsis thaliana* and the expression patterns of related genes. (**A**) qRT-PCR analysis of *VaHAESA* expression levels in transgenic *Arabidopsis thaliana*; (**B**) PCR detection of positive transgenic *Arabidopsis thaliana*; (**C**) phenotypic identification of *Arabidopsis thaliana* after inoculation with *H. arabidopsidis*: I. wild type; II, transgenic *Arabidopsis thaliana*; (**D**) the expression patterns of related genes in the pathogen-associated molecular patterns (PAMP)-triggered immunity (PTI) pathway. Values on vertical axes indicate the fold-change in gene expression, normalized to the expression levels of *AtACTIN*, *AtSAND*, and *AtUBQ*. The *x*-axes represent the time since inoculation. Error bars represent the standard deviation calculated from three replicates.

To characterize the physiological functions of *VaHAESA* involved in disease resistance, ten transgenic lines of *Arabidopsis* were obtained, and three transgenic lines exhibiting stable expression and improved disease resistance, designated “line2, line3, and line4” (confirmed through PCR and qRT-PCR, Figure 11A,B), were selected for further experiments. The results show that the expression level of *VaHEASA* was up to 40-fold higher than that of the *AtActin*gene in transgenic *Arabidopsis*. To determine the resistance of the transgenic plants, wild-type and transgenic *A. thaliana* plants were inoculated with *Hyaloperonospora arabidopsidis*. After infection, we analyzed the expression patterns of key genes in the PTI pathway. Our results showed that after 5 days of inoculation, *H. arabidopsidis* was abundant on wild-type leaves, while transgenic *A. thaliana* did not harbor the pathogen (Figure 11CI,CII). As key genes in the PTI pathway, the expression of MPK3 (mitogen-activated protein kinase 3), MPK6 (mitogen-activated protein kinase 6), SCD1 (stomatal cytokinesis-defective 1), BAK1 (BRI1 associated receptor kinase 1), and BIK1 (Botrytis-induced kinase 1) in the transgenic plants was substantially higher than in wild-type plants. These results showed that the expression of MAPKs and receptor-like cytoplasmic kinases (RLCKs) was 5-fold higher in transgenic plants than in wild-type plants at 2 h to 8 h after infection. The expression of these genes in wild-type *A. thaliana* was only slightly increased at 8 h after inoculation. These results clearly show that compared to wild-type plants, the transgenic plants exhibited an enhanced ability to defend against the invading pathogen.

## 3. Discussion

In recent years, it has become apparent that PRRs exist in the plasma membrane within intricate protein complexes resembling supramolecular structures and require numerous regulators to initiate and fine-tune plant immune responses [17,18,19]. In plants, the RLKs have been implicated in the prevention of self-pollination, pathogen responses, hormone perception, signal transduction, and plant development. These functions are divided into two categories [20]. One category consists of kinases involved in cell growth and development. For instance, some studies have shown that in *Arabidopsis*, LRR-RLK HAESA (HAE) and the peptide hormone IDA (inflorescence deficient in abscission) control floral organ abscission [21,22,23]. HAESA is a plasma membrane-associated protein with serine/threonine protein kinase activity. The other category includes RLK proteins involved in plant–pathogen interactions and defense responses [7]. However, the functional classification of RLK on the basis of structure may actually be more complex due to cross talk between disease and developmental pathways or due to recognition of multiple ligands by a signal receptor [24,25].

Our analysis of the conserved domain of *VaHAESA*, which contains an intracellular kinase domain, revealed that it belongs to the LRR-RLK family. These PRRs contain extracellular domains that allow MAMP/DAMP(microbe/damage—associated molecular patterns) perception [26]. LRR-type PRRs localize to the cell membrane and bind to proteins or peptides such as bacterial flagellin, EF-Tu, or endogenous PEP peptides [27]. Our results regarding the subcellular location of *VaHAESA* were consistent with the findings of previous studies. Interestingly, FLS2 forms a complex with the regulatory LRR-RLK BAK1 quasi-instantaneously upon flg22 perception, suggesting that FLS2 and BAK1 already exist in proximity to each other in the plasma membrane [28]. The paradigm of signaling activation by receptor kinases implies that ligand binding via the extracellular domain causes activation of the intracellular kinase domain and phosphorylation of substrates that contribute to intracellular signal transduction.

Therefore, *VaHAESA* could receive the signal through the extracellular LRR domain structure and transmit that signal with the help of the intracellular protein kinase domain. Recent transcriptome analysis of abscission zones from wild-type and *hae* mutants indicate that the IDA-HAE signaling module triggers cell wall-degrading and cell wall-remodeling genes that are necessary for the abscission process, in addition to genes commonly related to defense against bacteria and fungi [29]. Finally, the observed oxidative burst could also trigger the activation of pathogen defense genes because shedding exposes a fresh cell surface, which may be highly susceptible to pathogen infection.

Plant receptor kinases ectopically expressed in plant cells can be expected to be processed and localized correctly. RLK involved in signaling mainly recognize exogenous elicitor complexes that activate defense-related pathways. Recognition results in the formation of ROS, changes in ion flux, and rapid phosphorylation of the kinase domains. These effects all invoke the activation of the MAPK cascade and activation of defense-related genes [30]. It is also highly likely that the adaptors and coreceptors required for the activation of downstream signaling are present in heterologous cells and allow a functional signal output upon stimulation with appropriate ligands. There are indications that the signaling pathways involved in defense and development exhibit common features, for example, sharing MAPKs signaling components [31,32]. Many RLCKs serve as the core of PRRs and downstream defense systems [33]. In these RLCKs, BIK1 (Botrytis-induced kinase 1) is an important central component [34]. When plants are induced by PAMPs, the complex of BAK1 and PAMP phosphorylates BIK1, after which BIK1 dissociates from the complex and activates the downstream signal. Subsequently, the downstream reaction is accomplished by the MAPK signaling pathway [35]. Early research on parsley cells showed that the activation of elicitor-responsive MAPK, a homolog of AtMPK3, by a fungal elicitor results in the translocation of MAPK into the nucleus [31]. In *Arabidopsis*, AtMPK6 is activated by the bacterial flagellin peptide or by xylanase from the fungus *Trichoderma viride* [36]. These results suggested that MAPK might phosphorylate transcription factors that are involved in the plant defense response. Numerous studies have shown that PTI occurs in the early phase of plant defense [3]. Most PRRs are present at low concentrations in the plasma membrane, where the expression of these PRRs does not show any obvious changes in response to challenge by pathogenic bacteria. However, some studies have shown that PRRs may be upregulated after pathogenic invasion, thereby enhancing plant resistance [37]. Our qRT-PCR results indicated that *VaHAESA* expression was upregulated in the early stages of invasion after inoculation with a *P. viticola* strain, which is consistent with early immune responses to pathogenic challenge.

Previous research has shown that H_2_O_2_ is one of the earliest measurable indicators of PTI activity, representing a stable measure of the ROS produced in plants in response to pathogenic infection and PAMPs. This production of H_2_O_2_ is typically apoplastic but is subsequently associated with intracellular immunity-related pathways that regulate disease resistance, such as systemic acquired resistance (SAR) and PTI [38,39]. The transient expression of *VaHAESA* in the *V. vinifera* ‘Thompson Seedless’ variety resulted in a decrease in disease incidence, accumulation of H_2_O_2_, increased NO levels, and deposition of callose. These results indicate that *VaHAESA* enhances resistance to *P. viticola* in grapevines via the induction of resistance signaling and other molecular and cell wall modifications. Moreover, our results indicate that these phenomena, which occurred at the very early stages of infection in the leaves, were caused by the transient expression of *VaHAESA* after inoculation with *P. viticola*. Our observations of increased H_2_O_2_ levels further suggest a close relationship between ROS- and NO-signaling during the responses to pathogenic attacks [40,41]. Concerning the lag phase of NO generation, cryptogein, and chitosan (a deacylated derivative of chitin) induce NO production within two minutes, and specifically in the case of chitosan, NO production was shown to increase constantly until the last measured time point [42]. The ultimate outcome of PTI is the induction of resistance responses that prevent microbial colonization. Callose deposition is typically one of the late defense responses to pathogen invasion, with accumulation beginning approximately 16 h after the initiation of PTI [3]. Callose deposition is an important feature of plant immunity and is thought to reinforce the cell wall at fungal penetration sites to impede further infection [39,43,44]. In the present study, we showed that *VaHAESA* expression increased the deposition of callose in transgenic *V. vinifera* inoculated with *P. viticola*, suggesting that callose plays an important role in the PTI resistance of grapevines to downy mildew.

In conclusion, there is a large genotypic component in the resistance of grapevines to downy mildew, and defense reactions occur with variable timing and intensity. Several studies have indicated that increased production of ROS (superoxide radicals, 4 to 6 hpi) is followed by a hypersensitive response (6 to 8 hpi); subsequent increased activity of peroxidase in cells flanking the infection area and in the vascular tissue (10 to 12 hpi); and finally, increased production, accumulation, or conversion of phenolic compounds (12 to 15 hpi) [45,46]. The present study shows that *VaHAESA* acts as a PRR in grapevines that could initiate responses against pathogenic attacks; however, more detailed characterization of the genes and pathways involved is necessary to improve our understanding of the role of *VaHAESA* in the resistance of grapevines to downy mildew.

## 4. Materials and Methods

### 4.1. Plant Materials, Plasmopara viticola Strains, and Pathogen Infection

One-year old *V. amurensis* grapevines of the “Shuanghong” variety were maintained in a greenhouse under a 16:8 h light:dark cycle at 25 °C, with 85% relative humidity. The *Plasmopara viticola* strains “ZJ-1-1” and “JL-7-2” [13] were subcultured on *V. amurensis* leaf discs every 10 d at 22 °C under a 16:8 h photoperiod. The third to fifth unfolded leaves from the shoot apex of *V. amurensis* were inoculated with a suspension of 10^5^ *P. viticola* sporangia∙mL^−1^. Three leaves were pooled to represent one replicate, and three independent replicates were collected from each sample. Infected leaves were collected at 0, 0.5, 1, 2, 4, 6, 8, 12, 24, 48, 72, and 96 h post-infection (hpi). These samples were used for subsequent reverse transcription polymerase chain reaction (RT-PCR) experiments.

### 4.2. qRT-PCR

We measured the expression patterns of PRRs in the leaves of “Shuanghong” at different time points post-inoculation with P. viticola strains “ZJ-1-1” and “JL-7-2”. Total RNA was extracted from leaves using a modified CTAB method [47]. One microgram of total RNA was reverse transcribed into first-strand cDNA using a cDNA Synthesis Kit (TaKaRa Biotechnology, Dalian, China). The PRR genes were identified through transcriptome analysis [13]. Primers for qRT-PCR were designed using Beacon Designer ver. 8.10 (Premier Biosoft, Palo Alto, CA, USA). Vitis elongation factor 1-α (EF1-α), ubiquitin-conjugating enzyme (UBQ), and SAND family protein (SAND) were used as internal controls to normalize all data [48,49]. The fold-change in gene expression was estimated based on threshold cycles via the 2^−ΔΔCT^ method [50].

### 4.3. Cloning, Sequencing, and Phylogenetic Characterization of VaHAESA

The full open reading frames of *VaHAESA* genes were amplified from cDNA isolated from *V. amurensis* leaves inoculated with *P. viticola* “ZJ-1-1” using gene-specific primers (forward: 5ʹ-ATGTCGAAAACACCCCCACCTTCTG-3ʹ, reverse: 5ʹ-TCACACATTGGACGCAAAATTC-3ʹ). PCR amplification was performed in a final volume of 50 μL under the following conditions: initial denaturation at 98 °C for 3 min; followed by 34 cycles of 98 °C for 30 s, 55 °C for 30 s, and 72 °C for 1 min; with a final extension at 72 °C for 5 min (Phusion High-Fidelity PCR Kit; NEB). The amplification products were cloned into the expression vector pBI121. The putative VaRLK protein sequences were submitted to Pfam (available online: http://pfam.xfam.org) and SUPERFAMILY 2 (available online: http://supfam.org) to further characterize this gene.

Phylogenetic analyses were performed using 27 known LRR-RLKs from *Arabidopsis* and several HAESA or HAESA-like proteins from five different plants. Mega 5.05 software (Koichiro Tamura, et al., 2011) was employed to infer and align protein sequences with the default parameters [51]. A phylogenetic tree was constructed using the maximum-likelihood method, the JTT substitution model, and the “G + I rates among sites” model. Bootstrapping with 500 replicates was performed to assess the reliability of internal branches, and the nodes with bootstrap values greater than 50 were marked.

### 4.4. Agrobacterium-Mediated Transient Expression in Plants

To explore the function of the *VaHAESA* gene in disease resistance in grapevines, we transiently transformed *Vitis vinifera* variety “Thompson Seedless” plants with the expression vector carrying *VaHAESA* and the empty vector and then inoculated the transgenic plants with compatible *P. viticola* to test the effect of *VaHAESA* on resistance. The constructs and empty vectors were transformed into *Agrobacterium tumefaciens* “GV3101” according the manufacturer’s instructions (BC304-01, Biomed, Beijing, China). Agrobacterium-mediated transient expression in *Nicotiana benthamiana* was performed as previously described to assess localization [52], and *Agrobacterium*-mediated transformation of *Vitis vinifera* “Thompson Seedless” was performed using vacuum infiltration according to previously published methods [53].

### 4.5. Analysis of the Subcellular Localization of VaHAESA and Its Effect on Pathogen Infection

To verify that the expression of *VaHAESA* improved disease resistance in *V. vinifera*, we performed a microscopic examination of the leaves three days after inoculation. To analyze the subcellular localization of *VaHAESA* in *N. benthamiana*, the leaves were immersed in PBS buffer containing 5 mg·L^−1^ 4,6-diamidino-2-phenylindole (DAPI) for 10 min to stain the nuclei. Subsequently, leaf patches were mounted on microscope slides and observed using a Nikon C1 Si/TE2000E confocal laser-scanning microscope (Nikon, Minato, Tokyo, Japan).

To observe the development of *P. viticola* after leaf inoculation, *V. vinifera* ‘Thompson Seedless’ leaves from untransformed wild-type plants transiently expressing *VaHAESA* were collected at the corresponding time points. The leaves were stained with lactophenol-trypan blue (10 mL of lactic acid, 10 mL of glycerol, 10 g of phenol, and 10 mg of trypan blue dissolved in 10 mL of distilled water) following Keogh et al. [54]. For the analysis of callose, the leaves were treated according to the KOH-aniline blue fluorescence method [55]. Callose deposits were visualized under a UV filter using a fluorescence microscope and were counted using ImageJ 1.43U software (available online: https://imagej.nih.gov/ij/index.html).

The number of deposits was expressed as the mean of three different leaf areas. Under the microscope at a constant magnification, five fields of view were selected for each leaf, and the number of calli was counted. The average value was calculated for the final statistics.

### 4.6. Analysis of H_2_O_2_ and NO Levels in Transgenic Vitis vinifera Expressing VaHAESA

The detection of H_2_O_2_ was performed using a Hydrogen Peroxide Assay Kit (S0038, Beyotime, Shanghai, China). Each sample was ground to powder with liquid nitrogen, and 100 mg of the sample was then transferred to a 1.5 mL screw-cap tube containing 1.5 mL of lysate solvent. The tubes were shaken at a speed of 12,000 rpm at 4 °C for 5 min, after which the suspension was placed on ice. The lysate was used to dilute H_2_O_2_ to concentrations of 1, 3, 10, 30, and 100 µM, which were employed as standards. Then, 50 µL of the samples or standard products was added to a 96-well plate, and 100 µL of the peroxide detection reagent was added. After a mild shock, the 96-well plate was placed at room temperature for 30 min. The absorbance was detected at A560. The content of H_2_O_2_ in the sample was calculated according to the standard curve. The detection of NO was performed using the Total Nitric Oxide Assay Kit (S0023, Beyotime, Shanghai, China) according to the manufacturer’s instructions. First, 1 g of frozen leaf tissue was ground and then added to 50 µL of Griess Reagent I. The mixture was then heated for 5 min in a boiling water bath to denature the proteins, followed by centrifugation for 5 min at 12,000 *g*. The supernatants were subsequently collected, and 5 µL of 20 mM NADPH, 10 µL of FAD and 5 µL of nitrate reductase were added. After mixing, the samples were incubated at 37 °C for 30 min. Next, 10 µL LDH buffer and 10 µL LDH were added to the mixture, followed by incubation at 37 °C for 30 min. Finally, 50 µL of Griess Reagent I and Griess Reagent II were added to the mixture. A540 was determined after incubation for 10 min at room temperature (20–30 °C).

### 4.7. Screening and Identification of Transgenic Arabidopsis thaliana

To identify the function of the *VaHAESA* gene, the transformation vector 35S::*VaHAESA*-pBI121 was transformed into *Agrobacterium tumefaciens* “GV3101”. Using the floral dip transformation method [56], these strains were transferred to wild-type *Arabidopsis thaliana*. Transgenic plants were selected using 1/2 MS media containing 50 mg∙L^−1^ kanamycin and then transferred to soil (24 °C; 16/8 h light/dark). Screening was performed until stable homozygous T_3_ lines were obtained, and transgenic plants were subsequently tested for *VaHAESA* expression via PCR and qRT-PCR. Disease resistance in wild-type and transgenic *A. thaliana* was monitored through inoculation with *H. arabidopsidis*, and the detection of related genes was performed via qRT-PCR. *A. thaliana* was inoculated with a suspension of 10^5^ *H. arabidopsidis* sporangia∙mL^−1^. Three independent lines were collected from each sample. Infected leaves were collected at 0, 2, 4, 8, 12, 24, 48, and 72 h post-infection (hpi). These samples were used for subsequent qRT-PCR experiments. *AtACTIN*, *AtUBQ,* and *AtSAND* were employed as internal controls to normalize all data. The phenotypic observation of disease resistance was conducted at 5 dpi.
